# A Suspected Case of Kluver-Bucy Syndrome in an Adolescent Male Following SARS-CoV-2 Infection

**DOI:** 10.1192/bjo.2024.654

**Published:** 2024-08-01

**Authors:** Christina Barmpagianni, Kerenza MacDaid, Joanne Williams

**Affiliations:** Surrey and Borders Partnership NHS Foundation Trust, Surrey, United Kingdom

## Abstract

**Aims:**

We present a case of suspected Kluver-Bucy syndrome in an adolescent male, following a SARS-CoV-2 (Covid-19) infection. To the best of our knowledge, KBS has not been associated with Covid-19 before.

**Methods:**

A 15-year-old male with a background of autism spectrum disorder (ASD) was reviewed in a children and adolescent mental health outpatient clinic. The young person was non-verbal, and history was taken from his next of kin. In the last four weeks, he had developed acute onset hyperphagia with weight gain (88^th^ percentile for age), new onset physical and verbal aggression, and hyperorality, whereby the young person was exploring household objects with his mouth. A degree of hypersexuality was also noted in the form of rubbing and touching of the genital area.

There was no history of trauma or epilepsy; recent traveling or environmental change; psychosocial stressors or new medications, operations, or immunisations in the past year. The young person had a Covid-19 infection the month before the symptoms started. He was immunised against Covid-19 and this was the second time he contracted the infection, the first being 1 ½ years ago with full recovery.

The sudden onset of hyperphagia, aggression, hyperorality, and hypersexuality with the only known precipitating factor the recent Covid-19 infection, raised clinical suspicion for Kluver-Bucy syndrome. Six months later, the symptoms were milder but still present and no other cause had been identified. Due to ASD features, visual field testing, brain imaging, or routine blood tests were either not possible or required sedation and are being arranged with the support of his general practitioner.

**Results:**

Kluver-Bucy syndrome is a rare neurological disorder characterised by a distinct constellation of behavioural and cognitive symptoms resulting from bilateral lesions or dysfunction in the temporal lobes, particularly the amygdala. Patients often exhibit alterations in their behavioural repertoire, including hyperorality, hypersexuality, disinhibited behaviour, and visual agnosia. The presentation has been associated with temporal lobe infarcts, epilepsy, and herpes simplex encephalitis. The differential diagnosis was based on the fulfilment of clinical criteria for KBS, while other differentials included metabolic causes or behavioural manifestations related to ASD. Although investigations to explore other causes of symptoms are still being arranged, clinical suspicion for KBS was based on the presence of diagnostic criteria and the recent viral infection.

**Conclusion:**

Research is needed to identify potential associations between SARS and neuropsychiatric manifestations, while clinicians should be aware of the possibility of such complications.

